# Nuclear localization of the tyrosine kinase BMX mediates VEGFR2 expression

**DOI:** 10.1111/jcmm.14663

**Published:** 2019-10-23

**Authors:** Tingting Liu, Yonghao Li, Hong Su, Haifeng Zhang, Dennis Jones, Huanjiao Jenny Zhou, Weidong Ji, Wang Min

**Affiliations:** ^1^ The Center for Translational Medicine The First Affiliated Hospital Sun Yat‐sen University Guangzhou Guangdong China; ^2^ Zhongshan Ophthalmic Center Sun Yat‐sen University Guangzhou Guangdong China; ^3^ Department of Pathology and the Vascular Biology and Therapeutics Program Yale University School of Medicine New Haven CT USA; ^4^ Department of Pathology and Laboratory Medicine Boston University School of Medicine Boston MA USA

**Keywords:** angiogenesis, Bmx, promoter, Sp1, transcription, VEGFR2

## Abstract

Vascular endothelial growth factor receptors (VEGFRs) are major contributors to angiogenesis and lymphangiogenesis through the binding of VEGF ligands. We have previously shown that the bone marrow tyrosine kinase BMX is critical for inflammatory angiogenesis via its direct transactivation of VEGFR2. In the present study, we show that siRNA‐mediated silencing of BMX led to a significant decrease in the total levels of VEGFR2 mRNA and protein, without affecting their stability, in human endothelial cells (ECs). Interestingly, BMX was detected in the nuclei of ECs, and the SH3 domain of BMX was necessary for its nuclear localization. Luciferase assays showed a significant decrease in the *Vegfr2 (kdr)* gene promoter activity in ECs after BMX silencing, indicating that BMX is necessary for Vegfr2 transcription. In addition, we found that wild‐type BMX, but not a catalytic inactive mutant BMX‐K445R, promoted *Vegfr2* promoter activity and VEGF‐induced EC migration and tube sprouting. Mechanistically, we show that the enhancement of *Vegfr2* promoter activity by BMX was mediated by Sp1, a transcription factor critical for the *Vegfr2* promoter. Loss of BMX significantly reduced Sp1 binding to the Vegfr2 promoter as assayed by chromatin immunoprecipitation assays. Wild‐type BMX, but not a kinase‐inactive form of BMX, associated with and potentially phosphorylated Sp1. Moreover, a nuclear‐targeted BMX (NLS‐BMX), but not cytoplasm‐localized form (NES‐BMX), bound to Sp1 and augmented VEGFR2 expression. In conclusion, we uncovered a novel function of nuclear‐localized BMX in regulating VEGFR2 expression and angiogenesis, suggesting that BMX is a therapeutic target for angiogenesis‐related diseases.

## 1 INTRODUCTION

The human vasculature consists of blood and lymphatic vessels. The endothelium of these vessels plays critical roles in vessel functions such as proliferation, maintenance of immunity and metabolism.^1^ Identification of differential markers between blood and the lymphatic endothelium has recently allowed advanced study of the latter.^2^ However, specific proteins are common to both blood and the lymphatic endothelium, such as the surface receptors vascular endothelial growth factor receptors 2 and 3 (VEGFR2 and VEGFR3, respectively). VEGFR2 is critical for both angiogenesis and lymphangiogenesis.^3^, ^4^ The regulation of VEGFR2 activation and VEGFR2‐mediated angiogenesis signalling has been extensively investigated.^5^, ^6^, ^7^, ^8^ However, the mechanisms regulating VEGFR2 expression in endothelial cells (ECs) remain poorly characterized.

Bone marrow kinase in the X chromosome (BMX; also called endothelial/epithelial tyrosine kinase [ETK]) belongs to the TEC protein tyrosine kinase (TEC) family. BMX shares a common domain architecture with other TEC family kinases, including a pleckstrin homology (PH) domain, a TEC homology (TH) domain, Src homology domains (SH3 and SH2) and a kinase domain. Various proteins have been shown to interact with specific domains of BMX to facilitate intracellular signalling pathways.^9^ The PH domain is important for the binding of BMX to the plasma membrane, where it has been shown to interact with membrane lipids, leading to activation of signalling molecules. Followed by the PH domain is the TH domain, which does not contain the classical proline‐rich sequences found in other Tec family members.^9^ However, proline‐rich proteins have been shown to interact with the SH3 domain of BMX, leading to its activation.^10^ Adjacent to the SH3 domain is the SH2 domain, which binds phosphorylated tyrosine sites on proteins. At the C‐terminus of the BMX protein is the kinase domain. The kinase domain of TEC kinases is highly conserved. In addition to acting as a catalytic domain, the kinase domain of BMX serves as a site for proteins such as caveolin‐1 to bind.^11^ BMX activation is tightly regulated in the cellular processes such as migration, proliferation, survival and differentiation.^12^, ^13^, ^14^

In contrast to other TEC family members, BMX is broadly expressed in the endocardium, vascular endothelial and epithelial cells, and lymphoid and myeloid cell lineages. Because mice deficient in BMX do not have an obvious phenotype at steady state, BMX appears to be dispensable for embryonic and postnatal development in mice. However, we have recently shown that BMX can be up‐regulated in blood capillaries and LYVE1^+^ lymphatic vessels during endothelial remodelling.^15^, ^16^ We have reported that BMX is a downstream mediator of VEGFR2/3 signalling in HDLECs. BMX associates with and directly regulates VEGF‐A‐induced VEGFR2 activation.^16^ Importantly, BMX contributes to vascular remodelling and plays important roles in several other pathological models.^17^, ^18^, ^19^

The function of BMX as a signalling molecule, via its ability to interact with lipids and other proteins, has been extensively studied. The expression of BMX is regulated in vivo (and in vitro) by factors that are not well characterized. Interestingly, the cellular localization of BMX provides an additional layer of regulation and function for BMX. Most reports have shown that BMX is expressed in the cytoplasm, and BMX has also been categorized as a cytoplasmic tyrosine kinase. BMX localizes to different cellular locations such as at focal adhesions^20^ and in various organelles such as mitochondria^21^ and the nuclei^22^ of different cell types. Here, we report the dynamic localization of BMX in primary human ECs and show that BMX and its kinase catalytic activity are necessary for maximal VEGFR2 transcription.

## 2 MATERIALS AND METHODS

### 2.1 Cell culture

Human dermal lymphatic ECs (HDLECs) were purchased from Lonza (HMVEC‐dLyAd). HDLECs were cultured in EGM‐2 MV media in cell culture dishes coated with fibronectin (10 μg/mL) or 0.1% gelatin. Human umbilical vein ECs (HUVECs) were obtained from the Yale Skin Disease Research Center Endothelial Cell Facility under protocols approved by the Yale Human Investigation Committee. HUVECs were cultured in 0.1% gelatin‐coated dishes and were maintained in M199 media supplemented with 20% foetal bovine serum (FBS), 100 U/mL of penicillin, 100 μg/mL of streptomycin, 2 mmol/L l‐glutamine and 50 μg/mL of EC growth supplement. 293T cells were cultured in DMEM containing 20% FBS, 100 U/mL of penicillin and 100 μg/mL of streptomycin. COS7 cells were cultured in RPMI 1640 containing 20% FBS, 100 U/mL of penicillin and 100 μg/mL of streptomycin. All cell lines were cultured at 37°C in 5% CO_2_.

### 2.2 Cell stimulation

Recombinant human VEGF‐A was purchased from R&D Systems. HUVECs were serum‐starved for 16‐24 hours in endothelial basal media‐2 (EBM‐2, Lonza) before cells were stimulated at different time‐points with recombinant human VEGF‐A (50 ng/mL). Cycloheximide and actinomycin D were purchased from Sigma‐Aldrich and were used at a concentration of 10 μg/mL.

### 2.3 DNA constructs

Full‐length BMX or specified BMX domains were amplified by polymerase chain reaction (PCR) from BMX‐FLAG expression constructs.^12^, ^23^ The primers for full‐length BMX or specified BMX domains were designed to introduce SacI and KpnI restriction sites at either end of the cDNA. DNA products were directionally subcloned into the SacI‐KpnI multiple cloning sites downstream of green fluorescent protein (GFP) in the p‐EGFP‐C3 vector. A nuclear localization signal (NLS) PKKKRKV or a nuclear export signal (NES) LSPSLSPLSL was subcloned into the downstream of GFP in the GFP‐BMX‐WT vector to express GFP‐NLS‐BMX and GFP‐NES‐BMX, respectively. Positive clones were confirmed by restriction enzyme digestion, and the expression was confirmed by Western blotting. The plasmid pGL2, containing the firefly luciferase reporter gene under control of the –225 +268 region human VEGFR2 promoter, was used (kind gift from Dr Cam Patterson). The plasmid Sp1 was amplified by PCR from human cDNA, and DNA products were subcloned into the NotI‐MluI multiple cloning sites in the pLEX‐MCS vector.

#### 2.3.1 Chromatin immunoprecipitation assay

The chromatin immunoprecipitation (ChIP) assay was performed as described previously.^24^ The amount of Vegfr2 gene immunoprecipitated was quantified by real‐time PCR. The primers were 5′ GTCCAGTTGTGTGGGGAAAT 3′ (sense) and 5′ GAGCTGGAGCCGAAACTCTA 3′ (antisense), which amplified a 169‐bp region of the human Vegfr2 promoter containing Sp1 binding sites. The antibodies included anti‐Sp1 (#9389; Cell Signaling Technology), a positive control (Anti‐RNA Polymerase II) and a negative control (normal IgG) that were employed for each immunoprecipitation.

### 2.4 Cell transfection of siRNA and DNA

A small interfering (si) RNA for human BMX (RefSeq Number NM_001721) with the sequences 5′ GUCCCACAUUUCAGCAACUTT 3′ (sense) and 5′ AGUUGCUGAAAUGUGGGACTT 3′ (antisense), as well as a negative control siRNA with the sequences 5′ UUCUCCGAACGUGUCACGUTT 3′ (sense) and 5′ ACGUGACACGUUCGGAGAATT 3′ (antisense), were purchased from Sangon Biotech and were transfected into cells (20 nmol/L) by Oligofectamine following the manufacturer's protocols (Invitrogen). For cell transfection of DNA, 1 μg of mammalian expression plasmids was transfected into HDLECs and COS‐7 cells using Lipofectamine or Lipofectamine 2000 according to the manufacturer's protocol (Invitrogen). Cells were harvested at 24‐36 hours post‐transfection, and cell lysates were used for protein assays.

### 2.5 Western blotting

All protein samples were boiled for 10 minutes, resolved in 8%‐10% polyacrylamide gels and transferred to polyvinylidene difluoride membranes, followed by blocking with 5% milk diluted in phosphate‐buffered saline (PBS) containing 0.05% Tween‐20 (PBST). The membranes were then immunoblotted with the specified antibodies (1:1000 dilution in PBST) using horseradish peroxidase‐conjugated secondary antibodies (1:2000 dilution; GE Healthcare Life Sciences/Amersham Biosciences) and the enhanced chemiluminescence detection system (GE Healthcare Life Sciences, Amersham Biosciences).

### 2.6 Antibodies

Antibodies against VE‐cadherin were purchased from Santa Cruz Biotechnology. Anti‐Prox‐1, anti‐GFP and anti‐ZO‐1 antibodies were purchased from Abcam. Antibodies against BMX, VEGFR2, phosphor‐tyrosine (pY) and Sp1 were purchased from Cell Signaling Technology. BMXpY566 was from LifeSpan Biosciences, Inc (LSBio).

### 2.7 Immunofluorescence

Cells were grown on fibronectin‐coated glass chamber slides (VWR Scientific International). Cells transfected with GFP‐containing constructs were fixed with 2% paraformaldehyde (PFA) in PBS for 15 minutes at room temperature. The slides were then mounted onto glass slides with VECTASHIELD mounting medium with 4′,6‐diamidino‐2‐phenylindole (DAPI). GFP‐positive cells were analysed using a Zeiss Axiovert 200 fluorescence microscope or Leica Sp5 confocal microscope. Cells subjected to antibody staining were fixed with 2% PFA in PBS for 15 minutes at room temperature, permeabilized with 0.1% Triton X buffer, blocked in 1% foetal bovine serum diluted in PBS for 1 hour and stained 2 hours at room temperature or 4°C overnight using specified antibodies, followed by Alexa Fluor 488‐ or 594‐conjugated secondary antibodies (donkey anti‐goat, donkey anti‐rabbit, donkey antimouse or a combination for double immunofluorescence (IF) diluted at 1:300 in PBS; Invitrogen Molecular Probes).

### 2.8 RNA analysis

Real‐time PCR (qRT‐PCR) was performed with a Bio‐Rad C1000 Thermal Cycler. For mRNA analysis, total RNA was isolated from cells or lymph nodes using the RNeasy kit (Qiagen) according to the manufacturerʼs instructions. Reverse transcription was performed with a standard procedure (SuperScript First‐Strand Synthesis System; Invitrogen) with 0.5‐1 μg of total RNA. qRT‐PCR was performed with iQ SYBR Green Supermix in the C1000 Thermal Cycler (Bio‐Rad).

### 2.9 Immunoprecipitation

HUVECs were washed with cold PBS twice and were harvested in 700 μL of RIPA buffer (Beyotime Biotechnology) on ice. Cell lysates were then thawed on ice for 15 minutes and were subjected to centrifugation at 14 000 g at 4°C for 15 minutes; 600 μL of the supernatants was used immediately for IP. Next, 60 μL of lysate from each sample was used as the input, and the remaining lysate was equally separated into two parts for IP with anti‐GFP, anti‐Sp1 or anti‐IgG, separately. The lysates were then incubated with 5 μg of anti‐Sp1 or anti‐IgG and 20 μL of Protein A/G Magnetic Beads (Millipore) at 4°C overnight with rotation. Immune complexes were collected after each immunoprecipitation by a magnetic separator, followed by two washes with cold RIPA buffer. The immune complexes were then subjected to Western blotting as described above.

### 2.10 Migration Assay

Next, 4 × 10^4^ cells/well of HUVECs were cultured in a 96‐well plate in medium containing 0.5% foetal bovine serum overnight and were subjected to wound injury with a WoundMaker (Essen Bioscience). The cells were cultured in medium (0.5% foetal bovine serum) with or without VEGF (50 ng/mL) for the indicated times. The EC migration assay was determined by measuring the wound area using an IncuCyte Zoom cell migration invasion assay system (Essen Bioscience).

#### 2.10.1 Three‐dimensional bead sprouting assay

The spheroid sprouting assay was performed. HUVECs were infected with GFP or GFP‐BMX‐expressing lentivirus. ECs were seeded to Cytodex 3 microcarrier beads (Sigma) with the concentration of 400 cells per bead in Microvascular Endothelial Cell Growth Medium‐2 MV (EGM‐2, Lonza). These coated beads were embedded in fibrin gels. EGM‐2 medium containing fibroblasts (20 000 per well) was added to each well, and cultures were maintained for 4 days. Fluorescence images were captured with Axiovert 200 (Zeiss) at 10× magnification, and EC tube number and length were measured with NIH ImageJ.

### 2.11 Statistical analyses

All the data were expressed as means ± standard error of the mean (SEM). Statistical analysis was performed with two‐tailed Student's paired *t* test. Statistical significance for *P*‐values was as follows: *, *P* < .05; **, *P* < .01; ***, *P* < .001.

## 3 RESULTS

### 3.1 BMX regulates VEGFR2 expression in vascular ECs

**Figure 1:**
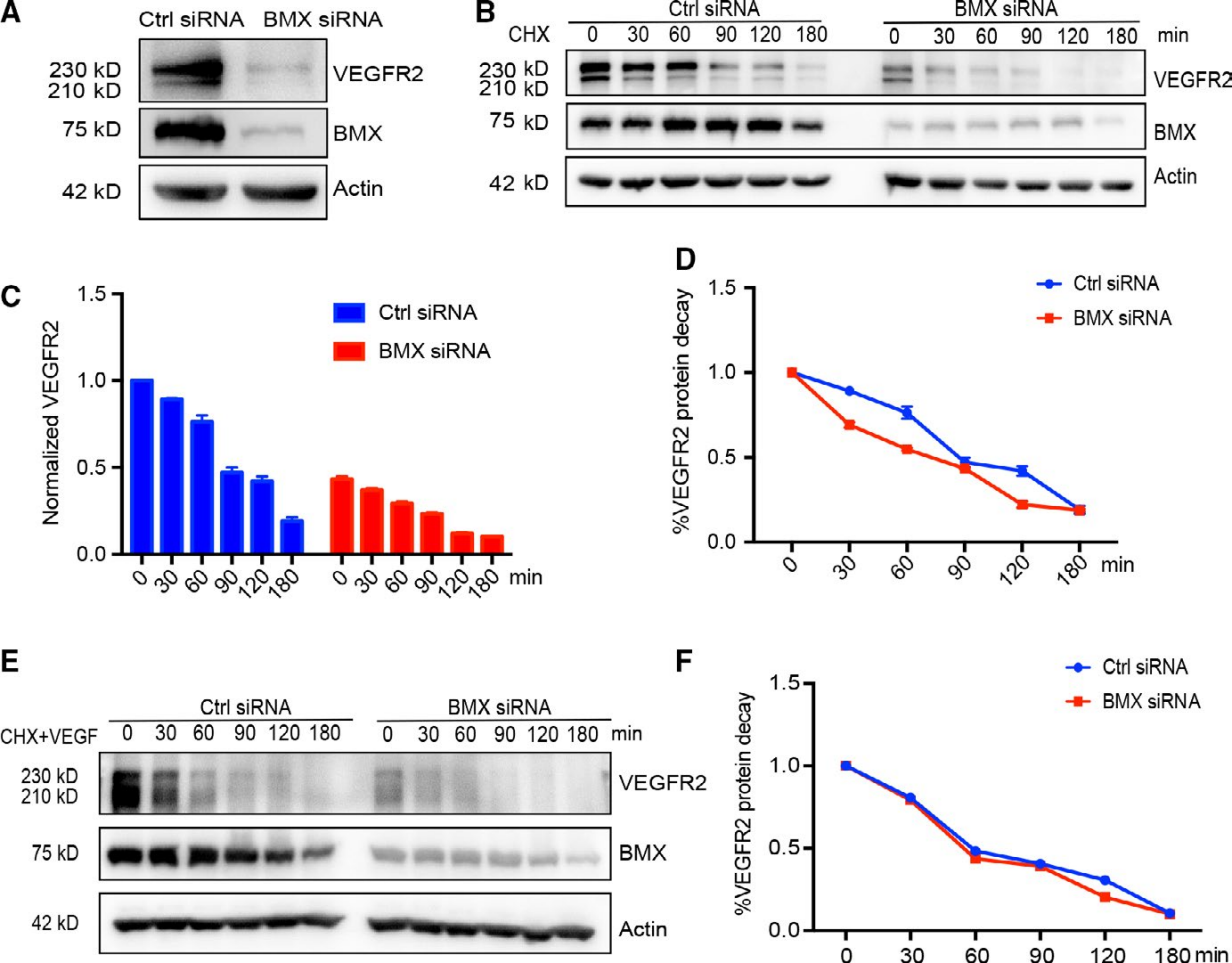
BMX regulates VEGFR2 expression but not VEGFR2 protein stability in ECs. HUVECs were transfected with human BMX siRNA or control siRNA (20 nmol/L) for 48 h. A, Total VEGFR2 and BMX proteins were determined by Western blotting with specific antibodies. β‐Actin was used as a loading control. B‐D, BMX siRNA on VEGFR2 stability. siRNA‐transfected HUVECs were incubated with cycloheximide (CHX, 10 μg/mL) for the indicated time‐points. Total VEGFR2 and BMX proteins were determined by Western blotting with specific antibodies. β‐Actin was used as a loading control (B). The protein bands in B were quantified by densitometry, and the relative VEGFR2 levels were presented by setting untreated control siRNA as 1.0 (C). Normalized VEGFR2 expression shows its decay rate (D). E‐F, BMX siRNA on VEGFR2 stability in the presence of VEGF‐A. siRNA‐transfected HUVECs were incubated with cycloheximide (CHX, 10 μg/mL) in the presence of VEGF‐A (50 ng/mL) for the indicated time‐points. VEGFR2 protein detection (E) and normalization (F) as described in B‐D. The data are means ± SEM from three independent experiments

VEGFR2 is expressed at low levels in most adult vessels but is strongly up‐regulated during neovascularization.^25^ We have previously shown that BMX and VEGFR2 were highly up‐regulated in the capillary endothelium in response to ischaemic injury. Interestingly, the expression and activation of VEGFR2 are BMX‐dependent, as evident by defective VEGFR2 induction in BMX knockout mice, but are increased in BMX transgenic mice.^15^ To determine the mechanism underlying BMX‐mediated VEGFR2 expression, BMX was knocked down by siRNA in human umbilical vein ECs (HUVECs) and human lymphatic ECs (HDLECs). We observed that siRNA‐mediated silencing of BMX led to a 40%‐80% decrease in the total levels of VEGFR2 protein in HUVECs (Figure 1A). To determine whether BMX affects the protein stability of VEGFR2, ECs were transfected with a control siRNA or BMX siRNA, followed by incubation with the protein biosynthesis inhibitor cycloheximide (CHX; 10 μg/mL). CHX treatment induced time‐dependent reduction of total VEGFR2 protein in both Ctrl siRNA and BMX siRNA‐transfected HUVECs (Figure 1B) and HDLECs (Figure S1A). Quantitative analyses indicated that the half‐life of VEGFR2 was not affected by BMX siRNA in HUVECs (Figure 1C‐D) or HDLECs (Figure S1A‐B). We further examined the VEGFR2 half‐life in the presence of VEGF‐A. BMX siRNA had no effect on the VEGFR2 half‐life in the presence of VEGF‐A (Figure 1E‐F). These data suggested that BMX regulates the VEGFR2 expression but not the VEGFR2 protein stability.

**Figure 2:**
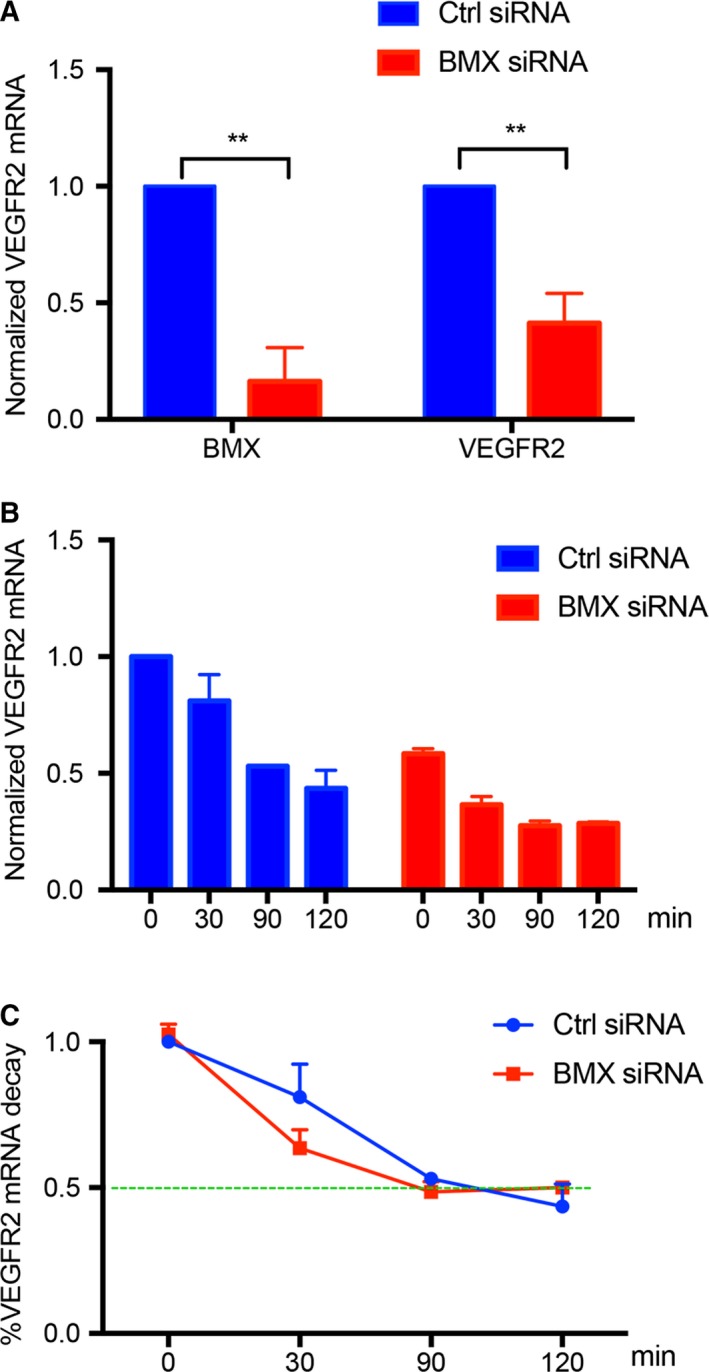
BMX regulates VEGFR2 expression at the transcriptional level in ECs. HUVECs were transfected with human BMX siRNA or control siRNA (20 nmol/L) for 48 h. A, siRNA‐transfected HUVECs were incubated with actinomycin D (10 μg/mL) for the indicated time‐points. VEGFR2 mRNA was assessed at the indicated time‐points by qRT‐PCR with normalization to β‐actin mRNA. B, The % decrease of VEGFR2 mRNA due to actinomycin D was quantified normalized to Ctrl siRNA at 0 min, using the time 0 of each respective group as the reference point. C, Normalized VEGFR2 mRNA levels. The data are means ± SEM from three independent experiments. **, *P* < .01

To determine whether BMX regulates VEGFR2 transcription, we analysed the effects of BMX siRNA on VEGFR2 mRNA expression in HUVECs by qRT‐PCR. In HUVECs with the knockdown of BMX by siRNA, the VEGFR2 transcript levels were decreased by 50%‐80% (Figure 2A). Because BMX has been implicated in transcript stability,^26^ we sought to determine whether this decreased VEGFR2 transcript level was due to reduced VEGFR2 transcript stability. To this end, the cells were incubated with the mRNA synthesis inhibitor actinomycin D (10 μg/mL). Actinomycin D led to a time‐dependent reduction of VEGFR2 mRNA in HUVECs (Figure 2B). The rate of VEGFR2 mRNA decay was similar between Ctrl siRNA and BMX siRNA‐treated HUVECs (Figure 2C). These data suggest that BMX regulates the VEGFR2 mRNA transcription, but not the VEGFR2 mRNA stability in ECs.

### 3.2 Dynamic localization of BMX in human ECs

**Figure 3:**
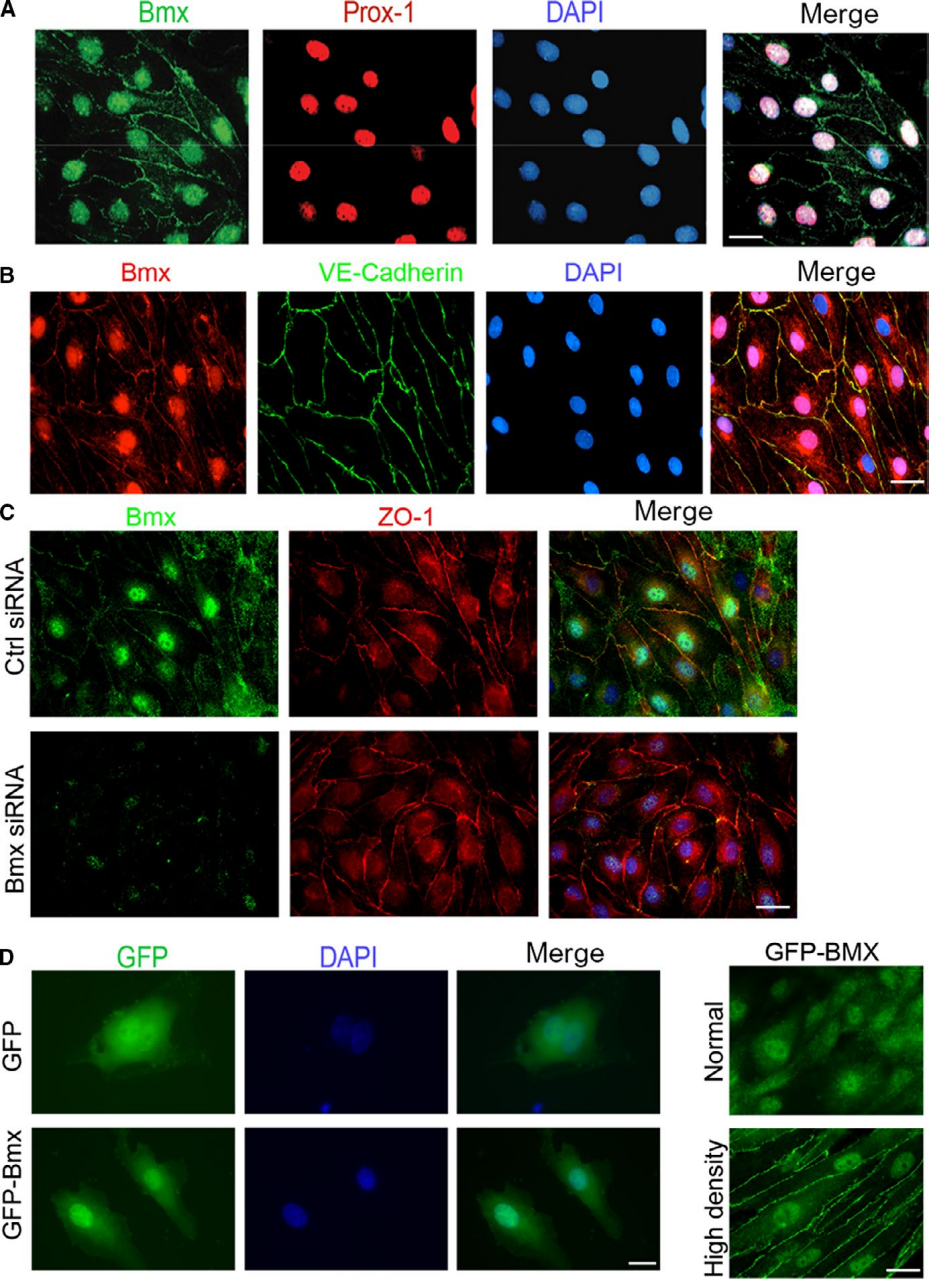
Localization of BMX in HDLECs. A, Confluent HDLECs were co‐stained with anti‐BMX and anti‐Prox‐1, followed by Alexa Fluor 488‐ and 594‐conjugated secondary antibodies (donkey anti‐goat and donkey anti‐rabbit, respectively). The merged images of BMX, Prox‐1 and DAPI are shown on the right. B. Confluent HDLECs were co‐stained with anti‐BMX and anti‐VE‐cadherin, followed by Alexa Fluor 594‐ and 488‐conjugated secondary antibodies (donkey anti‐goat and donkey antimouse, respectively). The merged images of BMX, VE‐cadherin and DAPI are shown on the right. 63× magnification images are shown for all images. C, HDLECs were transfected with human BMX siRNA or control siRNA (20 nmol/L) for 48 h. Confluent HDLECs were co‐stained with anti‐BMX and anti‐ZO‐1, followed by Alexa Fluor 488‐ and 594‐conjugated secondary antibodies (donkey anti‐goat and donkey antimouse, respectively). The merged images of BMX, ZO‐1 and DAPI from the control and BMX siRNA groups are shown on the right. D‐E, HDLECs were infected with lentivirus expressing GFP or GFP‐BMX. IF microscopy was used to visualize GFP or GFP‐BMX. Single cells (D), normal‐density (E, top) and high‐density cells (E, bottom) are shown. All experiments were repeated at least three times. Scale bar: 25 μm

Recent reports have shown that BMX can translocate to the cell nucleus.^22^ Because BMX does not have a canonical DNA‐binding domain, the potential of BMX to interact with transcription factors may explain how BMX could affect the transcription of VEGFR2. Therefore, we chose to carefully examine the localization of BMX in human lymphatic ECs (HDLECs). In subconfluent (sparse) seeding of HDLECs, BMX was restricted to the cytoplasm and at focal adhesions, which may be critical for its role in integrin and VEGFR2 activation.^9^, ^16^ Interestingly, we observed that, in confluent monolayer conditions, BMX was located at cell junctions and in nuclei. Nuclear staining was shown to localize with the transcription factor Prox‐1 in HDLECs (Figure 3A). Furthermore, BMX is co‐localized with the adherens junction protein VE‐cadherin (Figure 3B). Nuclear and adherens staining of BMX was specific because siRNA silencing of BMX reduced the staining intensity (Figure 3C).

To avoid reliance on antibodies, we investigated the cellular localization of full‐length human BMX as a chimeric protein fused to GFP. Similar to the endogenous BMX, GFP‐BMX in HDLECs was detected primarily in nucleus but some in the cytoplasm or cytoplasmic membrane in high‐density cultures (Figure 3D‐E). In addition, nuclear expression of BMX was found in HUVECs. The nuclear localization was specific to ECs because COS‐7 cells showed no nuclear accumulation of BMX (Figure S2). These data suggested that BMX can shuttle between different cellular compartments in ECs and that BMX localization is dependent on the cell type.

### 3.3 The SH3 domain of BMX is critical for BMX nuclear localization

**Figure 4:**
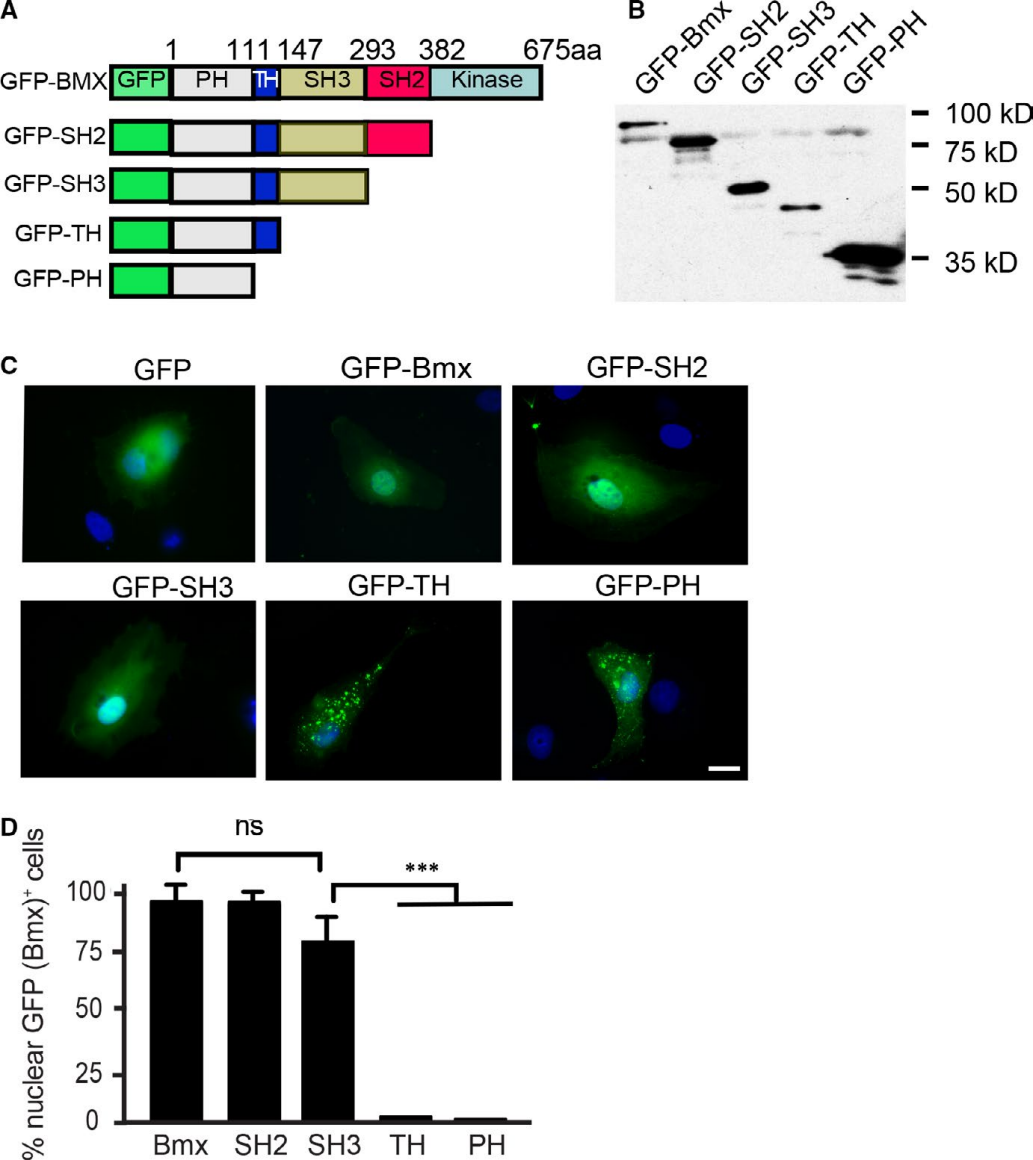
The SH3 domain of BMX is critical for BMX nuclear localization. A, Schematic diagram for BMX structural domains and expression constructs. GFP indicates green fluorescent protein; PH, pleckstrin homology domain; TH, TEC homology domain; K: kinase domain; aa, amino acids. Western blotting is shown in (B) using an anti‐GFP antibody to detect proteins. C, HDLECs were transfected with either 1.0 μg of GFP plasmid, GFP‐BMX full‐length plasmid (human BMX‐WT fused to an N‐terminal GFP tag), GFP‐SH2 plasmid (human BMX cDNA lacking the kinase domain fused to an N‐terminal GFP tag), GFP‐SH3 plasmid (human BMX PH, TH and SH3 domains domain fused to an N‐terminal GFP tag), GFP‐TH plasmid (human BMX PH and TH domains fused to an N‐terminal GFP tag) or GFP‐PH plasmid (human BMX PH domain fused to an N‐terminal GFP tag). IF microscopy was used to visualize the GFP tagged proteins. D, Quantification of nuclear GFP‐BMX^+^ cells. The data are means ± SEM from three independent experiments. One hundred GFP‐positive cells were counted for each group. ***, *P* < .0001. ns: non‐significant. Scale bar: 25 μm

Because BMX does not have a traditional nuclear localization signal (NLS), we manipulated the full‐length human BMX protein to determine which region of BMX was important for its nuclear localization. To this end, we generated truncations of the GFP‐BMX construct (Figure 4A‐B). GFP‐PH (GFP with the pleckstrin homology domain) showed punctate accumulations of BMX at the membrane and in the cytosol. A similar pattern was seen with GFP‐TH (GFP with the pleckstrin and Btk homology domains of BMX). Interestingly, once the SH3 domain was added (GFP‐SH3), cytoplasmic and nuclear localization of BMX was detected. Similar expression was found with GFP‐SH2 (GFP‐BMX without the kinase domain; Figure 4C‐D). Taken together, these data suggested that the SH3 domain of BMX is critical for its nuclear localization.

### 3.4 BMX regulates VEGFR2 expression and VEGF‐induced angiogenesis in a kinase‐dependent manner

**Figure 5:**
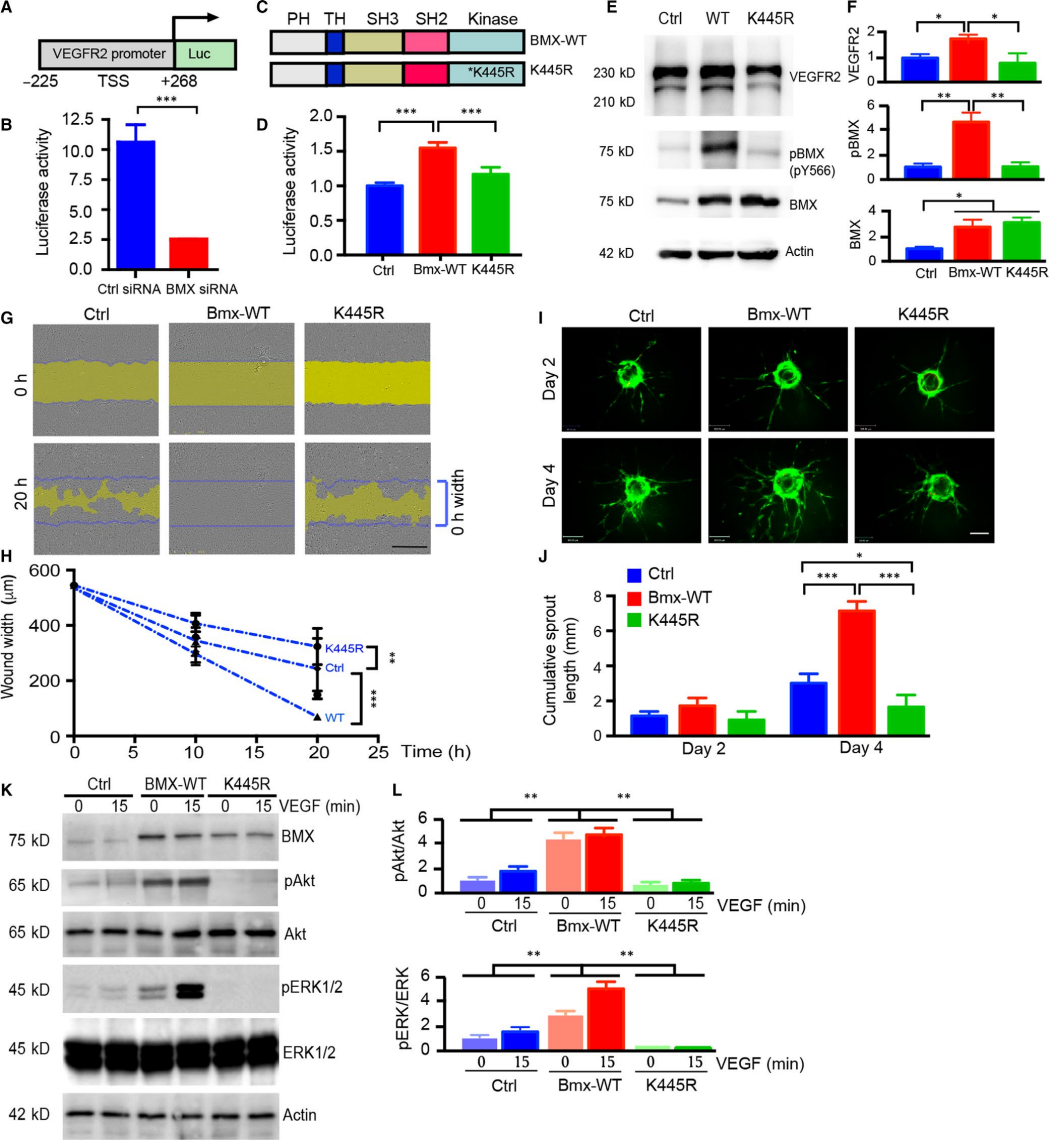
BMX regulates VEGFR2 transcription and VEGF‐induced angiogenesis in a kinase‐dependent manner. A, Schematic diagram for the Vegfr2 promoter‐luciferase reporter gene. −225 and +258 are the positions related to the transcription start site (TSS; +1). B, HDLECs were cotransfected with the Vegfr2 luciferase reporter, Renilla luciferase plasmid and either human BMX siRNA or control siRNA (20 nmol/L) for 48 h, and then, the dual‐luciferase assay was performed. For the dual‐luciferase assay, the firefly luciferase readout was normalized to that of Renilla luciferase. C, Schematic diagram of BMX‐WT and kinase‐dead mutant BMX‐K445R. D, HDLECs were cotransfected with the Vegfr2 luciferase reporter, Renilla luciferase plasmid and either vector control (Vector), BMX‐WT or kinase‐dead K445R‐BMX for 48 h, and then, the dual‐luciferase assay was performed. E, Overexpression of BMX‐WT, but not the kinase‐dead mutant BMX‐K445R in HUVECs, increases VEGFR2 expression. HUVECs were infected by lentivirus expressing vector control (Ctrl), BMX‐WT or BMX‐K445R. VEGFR2, BMX and phosphor‐BMX (pY566) were determined by Western blotting with indicated antibodies. F‐G, BMX‐WT promotes, while BMX‐K445R inhibits, VEGF‐induced EC migration. HUVECs were infected by lentivirus expressing vector control (Ctrl), BMX‐WT or BMX‐K445R. Cells were subjected to wound injury, followed by incubation for 10 h and 20 h in the presence of VGF‐A (50 ng/mL). F, Representative images of cell migration are shown. The initial wound gap (0 h) is indicated by blue lines at 20 h images, while the remaining gaps are indicated by yellow areas. G, Quantitation of EC migration. The average wound width was quantified, n = 3. Scale bar: 1 mm. H‐I, 3D spheroid sprouting assay. GFP, GFP‐BMX‐WT‐ or GFP‐BMX‐KR‐expressing HUVECs were coated with microbeads, embedded in fibrin gels and grown in EGM‐2 medium for 4 d. A representative image of 10 beads for each sample is shown (H). Scale bar: 100 μm. Quantifications of sprout number, sprout length are shown in I, n = 10. J, BMX‐WT promotes, while BMX‐K445R inhibits, VEGF‐induced signalling. HUVECs were infected by lentivirus expressing vector control (Ctrl), BMX‐WT or BMX‐K445R. Cells were treated with VEGF‐A (50 ng/mL) for 15 min, and VEGFR2 downstream signalling was determined by Western blotting with indicated antibodies, n = 3. Normalized p‐Akt:total Akt and p‐ERK1/2:total ERK1/2 were quantified by taking untreated Ctrl as 1.0. All data are means ± SEM from three independent experiments. *P < .05, **P < .01 and ***P < .001 by unpaired two‐tailed Student's *t* test

We reasoned that nuclear BMX might regulate VEGFR2 expression at the transcriptional level by directly enhancing Vgefr2 promoter activity. To address this possibility, we employed the luciferase reporter assay to determine the effect of BMX on the transcriptional activity of the Vegfr2 gene promoter (Figure 5A). The Vegfr2 promoter is located within −716 to +296 bp relative to the transcription start site of the VEGFR2 gene, and the short fragment (−225 to +268 bp) contains the core promoter activity.^27^ The Vegfr2 promoter‐based luciferase gene constructs were transiently transfected into ECs in the presence of Ctrl or BMX siRNA. The Vegfr2 gene promoter activities were significantly lower in HUVECs with BMX siRNA than in HUVECs with Ctrl siRNA, as determined by the luciferase activity assay (Figure 5B). Several studies have found that BMX tyrosine kinase activity has diverse functions under different conditions.^19^, ^28^ Next, we assessed whether BMX kinase activity was required for Vegfr2 promoter activation by rendering BMX catalytically inactive by mutating its ATP co‐ordination site (K445R; Figure 5C). Transient transfection of wild‐type BMX (WT) into HUVECs resulted in a significant increase in the Vegfr2 promoter activity, but the kinase‐dead BMXK445R mutant abolished Vegfr2 promoter activation (Figure 5D).

Because VEGFR2 expression is important for EC angiogenesis, we determined the role of BMX kinase activity in VEGF‐induced angiogenesis. To this end, HUVECs were infected by lentivirus expressing control vector (Ctrl), BMX‐WT and BMX‐K445R. Overexpression BMX‐WT, but not BMX‐K445R, induced auto‐phosphorylation at the tyrosine site 566 as determined by the p‐BMX (Y566)‐specific antibody.^16^ Similar to the effects of BMX on the Vegfr2 *promoter* activity, BMX‐WT increased, where BMX‐K445R mutant reduced, the endogenous VEGFR2 protein expression (Figure 5E with quantification in 5F).

Effect of BMX‐WT and BMX‐K445R on VEGF‐induced EC migration, a critical step for angiogenesis, was examined. HUVECs were starved in medium with 0.5% FBS overnight, followed by the wound healing assay in the presence of VEGF‐A (50 ng/mL). The effects of BMX‐WT and BMX‐K445R expression on VEGF‐induced HUVEC migration rates were determined by measuring the wound width confluent rates. BMX‐WT expression promoted VEGF‐induced (+VEGF) EC migration. By contrast, BMX‐K445R expression inhibited VEGF‐induced EC migration (Figure 5G‐H). We further determined the effect of BMX‐WT and BMX‐K445R on EC tube formation. To this end, we performed a 3D spheroid sprouting assay in which ECs were coated onto Cytodex beads followed by embedding in fibrin gels.^29^ Fibroblasts cultured on top of the gel promoted optimal sprouting and tube formation (Figure 5I). Quantitative analyses indicated that the cumulative sprout length was increased by BMX‐WT but attenuated by BMX‐K455R (Figure 5J). To define the underlying mechanism by which BMX‐K455R inhibited VEGF responses, we examined the effects of BMX‐K445R on the VEGFR2 signalling. As shown in Figure 5K with quantification in 5L, BMX‐K445R reduced VEGF‐induced signalling compared to Ctrl, including p‐Akt and p‐ERK1/2. These data indicate that BMX‐445R may function as a dominant negative form. Taken together, these results demonstrated that the kinase activity of BMX is not only required for VEGFR2 expression but also involved in VEGF‐induced angiogenesis.

### 3.5 BMX is critical for Sp1 transcriptional factor binding to the Vegfr2 promoter

**Figure 6:**
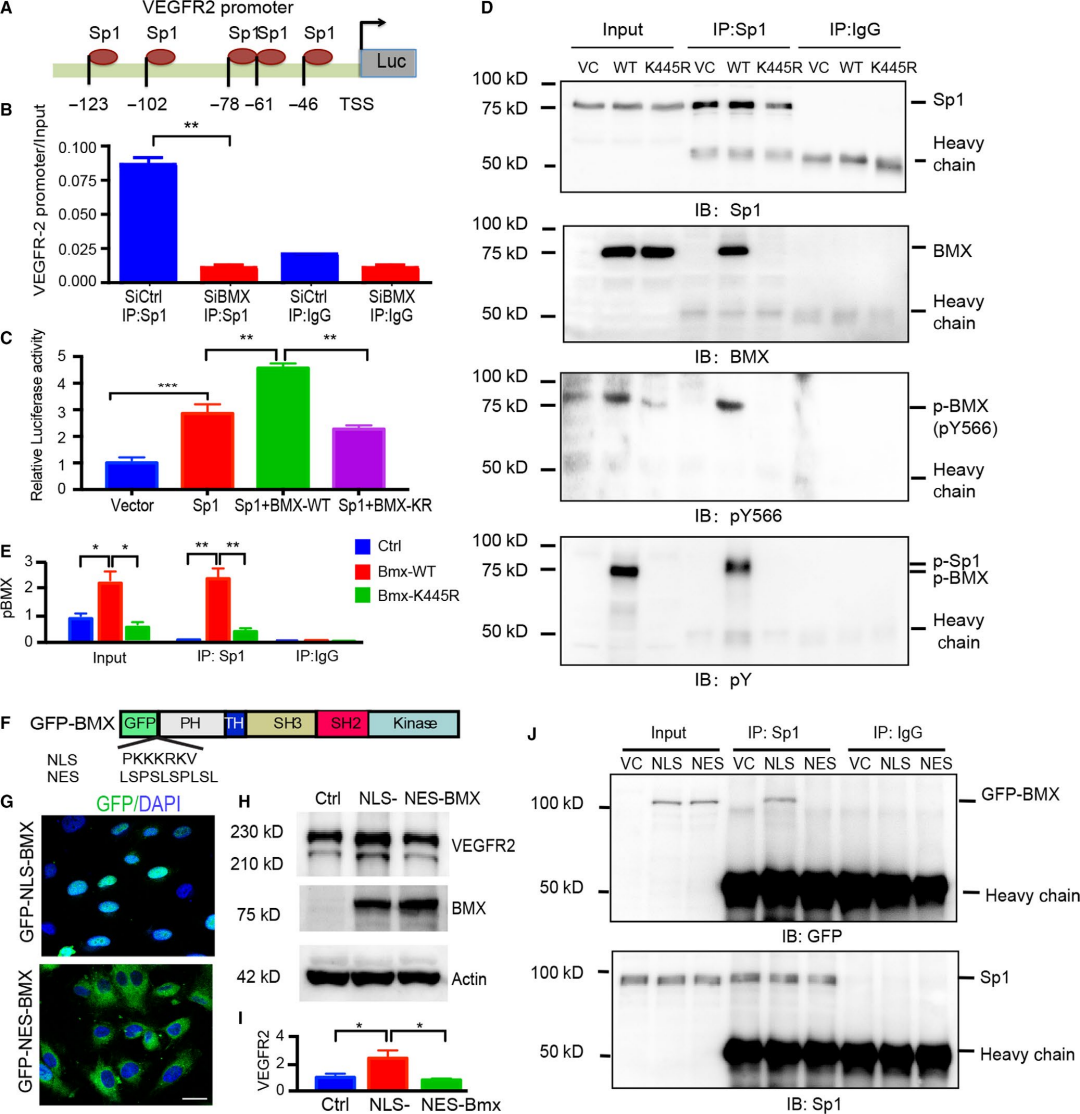
Active BMX interacts with Sp1 in the nucleus and facilitates Sp1 binding to the Vegfr2 promoter. A, Schematic diagram for the Sp1 binding sites located on the Vegfr2 promoter. −123 to −46 are positions related to the transcription start site (TSS; +1). B, BMX promotes Sp1 binding to the Vegfr2 promoter. HDLECs were transfected with human BMX siRNA or control siRNA (20 nmol/L) for 48 h. ChIP assay was then performed with Sp1 antibody. An Sp1 binding region of the Vegfr2 promoter was used as a primer for quantitative PCR. C, HUVECs were cotransfected with a Vegfr2 reporter (−123 to +1), Renilla luciferase plasmid and either vector control (Vector), BMX‐WT or kinase‐dead K445R‐BMX alone or together with Sp1 for 48 h. The dual‐luciferase assay was then performed. The firefly luciferase readout was normalized to that of Renilla luciferase. The data are means ± SEM from three independent experiments. **, *P* < .01; ***, *P* < .0001. D‐E, HUVECs were infected by BMX‐WT or BMX‐K445R lentivirus. The nuclear fractions were isolated, and the association of BMX with Sp1 was determined by immunoprecipitation with anti‐Sp1 antibody, followed by immunoblotting (IB) with anti‐Sp1, anti‐BMX, anti‐BMXpY566 and anti‐phosphor‐tyrosine (anti‐pY). IgG was used to a control. Quantification of p‐BMX was presented in (E). F‐G, Location of NLS‐BMX and NES‐BMX. F, Schematic diagram of GFP‐NLS‐BMX and GFP‐NES‐BMX. Insertion of a NLS (PKKKRKV) or NES (LSPSLSPLSL) is indicated. G, HUVECs were infected by lentivirus expressing GFP‐NLS‐BMX or GFP‐NES‐BMX, and location of BMX was visualized under fluorescence microscope. H‐J, NLS‐BMX, but not NES‐BMX, increases VEGFR2 and binds to Sp1. HUVECs were infected by lentivirus expressing vector control, GFP‐NLS‐BMX or GFP‐NES‐BMX. VEGFR2 expression was determined by Western blotting (H) with quantification in (I), and association of BMX with Sp1 was determined by co‐immunoprecipitation (J)

It was reported that transcriptional factor Sp1 binds to the Vegfr2 proximal promoter and regulates its activity.^30^, ^31^ We performed the chromatin immunoprecipitation (ChIP) assay to determine whether BMX affects the binding of Sp1 to the Vegfr2 promoter region. We chose a region of the human Vegfr2 proximal promoter that contains five Sp1 binding sites between −158 bp and +1 relative to the transcription start site (Figure 6A). ECs were immunoprecipitated with control IgG or Sp1. An isotype IgG was used as a negative control for immunoprecipitation. The GAPDH gene promoter was used as a negative control. The Sp1 binding region of the Vegfr2 promoter was used as a primer for quantitative PCR. Relative to control IgG, Sp1 immunoprecipitation showed higher binding of Sp1 to the Vegfr2 promoter. Moreover, knockdown of BMX led to significantly decreased association of Sp1 with the Vegfr2 promoter (Figure 6B). We then examined whether BMX affects Sp1‐mediated Vegfr2 transcription using a reporter gene driven by the Vefgr2 promoter (−158 bp to +1, containing the five Sp1 sites. We co‐expressed BMX‐WT or BMX‐K445R with Sp1 or Sp1 alone in ECs. Sp1 alone activated the Vegfr2 promoter; BMX‐WT promoted, but BMX‐K445R inhibited, Sp1‐mediated Vegfr2 promoter activation (Figure 6C). These results suggested that BMX kinase activity is necessary for the maximal transcriptional activity of the Vegfr2 gene.

We hypothesized that nuclear BMX could directly associate with Sp1 in the nucleus to facilitate Sp1 binding to the Vegfr2 promoter. To test our hypothesis, BMX‐WT or BMX‐K445R was expressed in HUVECs, and the association of BMX and Sp1 was determined by co‐immunoprecipitation with anti‐Sp1 followed by immunoblotting with anti‐BMX. Sp1 co‐immunoprecipitation brought down BMX‐WT but not BMX‐K445R, and BMX‐WT could be detected by both total BMX and BMXpY566. Moreover, phosphorylated Sp1 was detected in the presence of BMX‐WT but not in the presence of BMX‐K445R as detected by the immunoblotting of anti‐phosphotyrosine (Figure 6D with quantification in 6E for p‐BMX). These results suggested that BMX could associate with and phosphorylate Sp1.

To determine whether the nuclear localization of BMX mediates the VEGFR2 expression, a nuclear localization signal (NLS) PKKKRKV or a nuclear export signal (NES) LSPSLSPLSL was subcloned into the downstream of GFP in the GFP‐BMX‐WT vector to express GFP‐NLS‐BMX and GFP‐NES‐BMX, respectively (Figure 6F). Expression and localization analyses in ECs indicated that GFP‐NLS‐BMX was accumulated in the nucleus whereas GFP‐NES‐BMX was exclusively in the cytoplasm (Figure 6G). We then determined effects of GFP‐NLS‐BMX and GFP‐NES‐BMX on VEGFR2 expression. Western blotting showed that GFP‐NLS‐BMX, but not GFP‐NES‐BMX, promoted the VEGFR2 expression in ECs (Figure 6H with quantification in 6I). Consistently, GFP‐NLS‐BMX, but not GFP‐NES‐BMX, is associated with Sp1 in a co‐immunoprecipitation assay (Figure 6J). These results demonstrate that BMX nuclear localization is critical for the Sp1 binding and for the Sp1‐mediated VEGFR2 expression.

## 4 DISCUSSION

We have previously shown that BMX and VEGFR2 were highly up‐regulated in the ischaemic hindlimb.^15^ Interestingly, the expression and activation of VEGFR2 are BMX‐dependent (ie defective in BMX‐KO but increased in BMX‐SK‐Tg mice). These results suggested that BMX is not only a downstream effector (activated by VEGFR2) but also an upstream activator of VEGFR2. This phenomenon has been reported for BMX and VEGF signalling.^13^, ^32^ Consistent with a previous microarray analysis,^33^ we observed that BMX is critical for the gene expression of proinflammatory and proangiogenic molecules, likely via VEGFR2‐dependent pathways.^15^, ^23^ However, the exact mechanism by which BMX expression/activation in ECs regulates the gene expression of these molecules is unknown.

**Figure 7:**
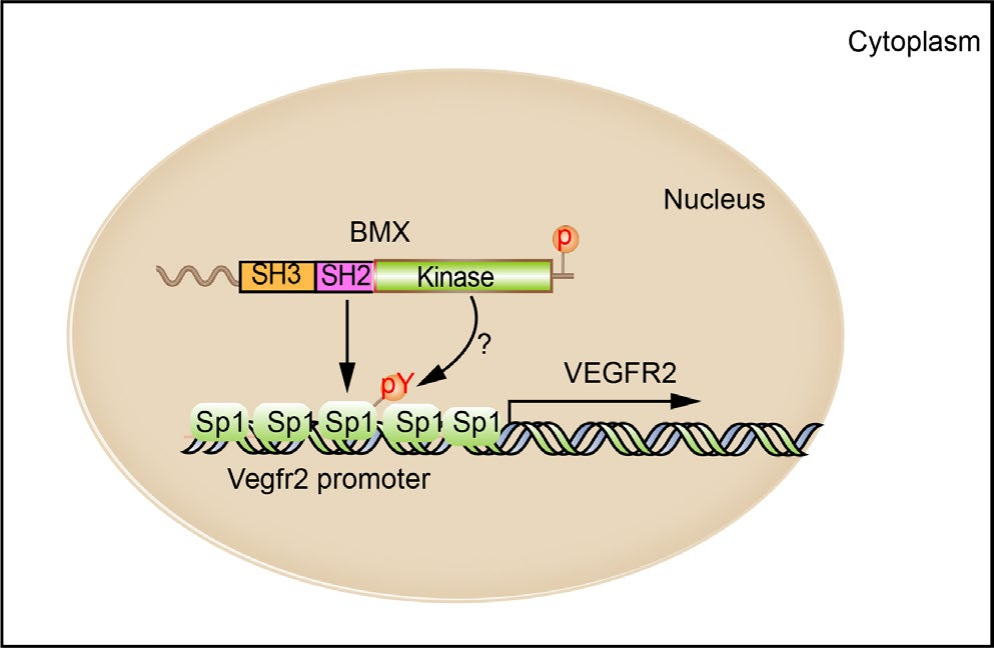
Schematic model of BMX regulates VEGFR2 transcription by interaction with Sp1. The SH2/SH3 domain of BMX confers its nuclear localization in ECs. Nuclear BMX in an active form interacts with (and possibly phosphorylates) Sp1 to facilitate the recruitment of Sp1 to the VEGFR2 promoter and its transcription in ECs

In the present study, we demonstrated the unexpected localization and function of BMX in ECs. Specifically, BMX is detected in the nuclei of ECs in monolayer culture. Furthermore, BMX is necessary for the maintenance of VEGFR2 expression at the transcriptional level. Our study has uncovered a novel function of BMX in regulating VEGFR2 expression and angiogenesis. These findings raise many questions. (a) What is the signal that allows BMX to enter the nucleus? This appears to be independent of cell proliferation because both Ki‐67‐negative and Ki‐67‐positive HDLECs can express nuclear BMX (data not shown). In addition, stimulation of HDLECs with VEGF‐A and VEGF‐C does not influence the nuclear localization of BMX. (b) Because BMX does not have a classical NLS, which protein(s) are responsible for the entry of BMX into the nucleus? It is possible that the SH3 domain of BMX can inhibit the plasma membrane localization of BMX by masking the PH domain. Concomitantly, a proline‐rich protein in ECs may bind to the SH3 domain of BMX, facilitating its nuclear localization. Alternatively, BMX is translocated into nucleus by forming a complex with other nuclear proteins. It is known that BMX via its PH domain associates with the FERM domain of focal adhesion kinase (FAK) in the integrin signalling.^20^ Interestingly, a recent study indicates that FAK is also translocated into the nucleus where it is detected in the RNA polymerase II complex associated with the Vegfr2 promoter, regulating the VEGFR2 transcription.^34^ It is plausible that the BMX‐FAK protein complex translocates to the nucleus to drive the Vegfr2 gene expression. This needs to be further examined. (c) Once BMX is in the nucleus, what function does it serve and what are its interacting partners? Here, we show that activated BMX directly interacts with Sp1 in the nucleus, and this interaction enhances the recruitment of Sp1 to the VEGFR2 promoter to promote transcription (Figure 7). A prior study reported that BMX was found in the nuclei of cells from human prostate tumour samples.^22^, ^35^ In these studies, BMX expression was associated with increased androgen receptor phosphorylation. The same group reported that the silencing of BMX significantly decreased histone H3 acetylation at lysine 18 (K18), which is correlated with attenuated gene transcriptional activation.^10^ It is also possible that BMX may interact with histones, thereby rendering the transcriptional changes. (d) How do different cell types control the nuclear entry of BMX? The nuclear localization of BMX appears to be specific to ECs (this study) and tumour cells. It would be interesting to determine the specific properties of these cells that allow the nuclear localization of BMX. More work is needed to address the aforementioned questions.

Other reports have shown that BMX is important for the mRNA stability of genes such as interleukin (IL)‐6 and VEGF in fibroblasts.^26^ This finding contrasts with ours, suggesting that BMX is not critical for the mRNA stability of VEGFR2 in ECs. The differences may be due to cell type–specific effects. It has also been reported that BMX is sufficient for the activation of VEGF enhancer/promoter activity, which is regulated by both ERK and PI3K pathways in ECs.^32^ In our study, we first found the kinase‐dependent transcription‐promoting function of BMX by the overexpression of BMX‐WT, but not kinase‐dead K445R mutant BMX, increased VEGFR2 gene transcripts.

Each TEC family kinase shows a unique pattern of subcellular localization, residing in the cytosol or at the plasma membrane, where they phosphorylate their major substrates. Interestingly, several studies have indicated that TEC kinases may play important roles in the nucleus. In CD3‐stimulated T cells, the Itk protein is found in the nucleus. In this study, the nuclear localization was dependent on binding to karyopherin alpha (Rch1α), a nuclear transporter. This interaction was important for IL‐2 production by T cells.^36^ In addition, Itk associated with and led to the phosphorylation of T‐bet, a nuclear transcription factor that regulates IFN‐γ transcription.^37^ The Rlk protein is expressed in T cells as two isoforms. The larger 58‐kD isoform is cytoplasmic and localizes to lipid rafts via its cysteine string motif. The shorter 52‐kD isoform does not contain a cysteine‐string motif and localizes to the nucleus via its nuclear localization sequence.^38^ Not much is known about the function of nuclear Rlk. Finally, the ability to shuttle between the cytoplasm and nucleus has been observed for Btk.^39^ The nuclear observations for other TEC family kinases add intrigue to the observation of BMX nuclear localization. The data from our study identify a nuclear target for BMX. Because the loss of BMX affects Sp1 binding to the VEGFR2 promoter, BMX‐WT, but not the kinase‐dead mutant BMX‐K445R, promotes VEGFR2 promoter transcription together with Sp1. Our efforts to map the interaction between Sp1 and active BMX suggest that BMX kinase activity plays a pivotal role in Sp1 regulation of VEGFR2 transcription. Consistently, BMX‐WT, but not the BMX‐K445R mutant, enhances VEGF‐induced EC migration and tube formation. Of note, BMX‐K445R expression ECs show slower migration and less tube formation than the control cells, suggesting that BMX‐445R may function as a dominant negative form.

Several studies have found that BMX tyrosine kinase activity has diverse functions under different conditions. In pathological cardiac hypertrophy development, inactivation of BMX abolished Ang II‐induced cardiac hypertrophy in mice. The effect of BMX on cardiac hypertrophy is mediated via the crosstalk between ECs of the coronary vasculature and cardiomyocytes.^28^ In another study, BMX kinase activity was found to be indispensable for IL‐8 promoter activation. IL‐8 promoter activation was completely abolished for all kinase‐dead BMX mutants.^19^ Our current study supports that BMX kinase activity is indispensable for the regulation of VEGFR2 expression. We have previously revealed that BMX kinase activity plays an important role in TNF‐induced EC angiogenesis 12 and VEGF‐induced lymphangiogenesis.^16^ With the development of specific BMX tyrosine kinase inhibitors, the inhibition of BMX may represent a feasible approach to synergistically attack tumour cells and tumour‐associated angiogenesis.^17^

## CONFLICT OF INTEREST

The authors confirm that there are no conflicts of interest.

## AUTHOR CONTRIBUTIONS

TL, YL, HS, ZH, DJ, HJZ, and WM designed the study, performed the experiments and analysed the data; TL and WM wrote the paper; and DJ and HJZ edited the paper.

## Data Availability

All other data supporting the presented findings are available from the corresponding author upon request.
