# Supplementary material for: Nuclear localization of the tyrosine kinase BMX mediates VEGFR2 expression
Source: J Cell Mol Med. 2019 Oct 23;24(1):126–38. doi: 10.1111/jcmm.14663 (PMC6933376; doi:10.1111/jcmm.14663)
Supplement: Supplementary file 1 [file JCMM-24-126-s001.pdf]

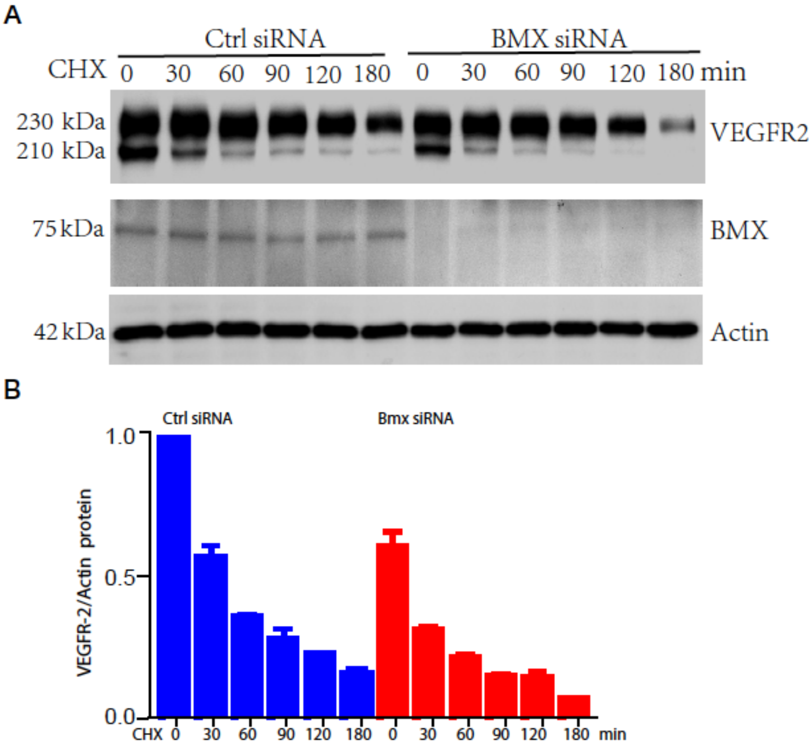

Supplemental Figure 1. BMX regulates VEGFR2 expression in HDLECs. Human lymphatic ECs (HDLECs) were transfected with human BMX siRNA or control siRNA (20 nmol/L) for 48 h. siRNA-transfected HUVECs were incubated with cycloheximide (CHX, 10 µg/ml) for the indicated time points. **A**. Total VEGFR2 and BMX proteins were determined by Western blotting with specific antibodies. β-Actin was used as a loading control. **B.** The protein bands in B were quantified by densitometry, and the relative VEGFR2 levels were presented by setting untreated control siRNA as 1.0. The data are means± SEM from three independent experiments.

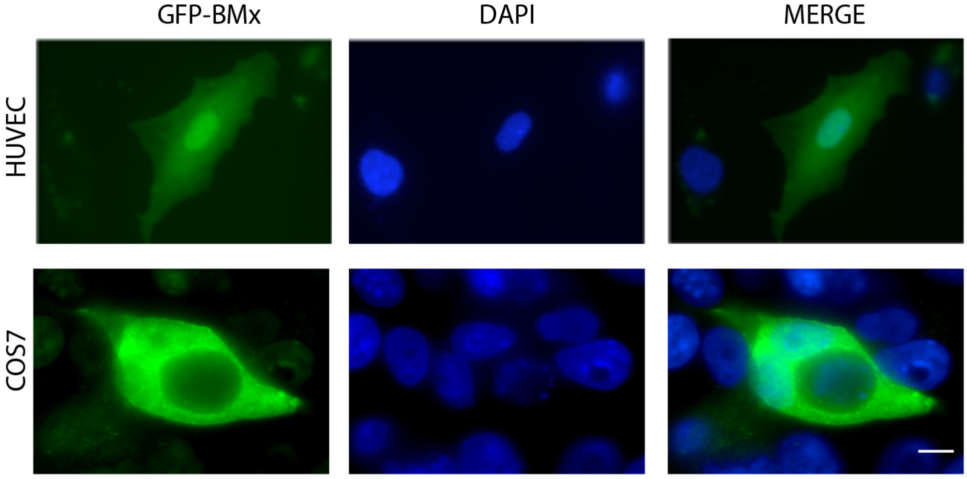

Supplement Figure 2. Localization of GFP-BMX in HUVECs and COS-7 cells. GFP-BMX was transfected into HUVECs or Cos7 for 48 h. GFP-positive cells were imaged under a fluorescence microscope. The merged images of GFP and DAPI are shown on the right. 63× magnification images are shown for all images. Experiments was repeated at least three times. Scale bar: 25 μm.
